# A rich internet application for remote visualization and collaborative annotation of digital slides in histology and cytology

**DOI:** 10.1186/1746-1596-8-S1-S26

**Published:** 2013-09-30

**Authors:** Raphaël Marée, Benjamin Stévens, Loïc Rollus, Natacha Rocks, Xavier Moles Lopez, Isabelle Salmon, Didier Cataldo, Louis Wehenkel

**Affiliations:** 1 GIGA-Systems Biology & Chemical Biology, GIGA-R, University of Liège, Belgium; 2 Systems and Modeling, Montefiore Institute, University of Liège, Belgium; 3 GIGA Bioinformatics Core Facility, GIGA, University of Liège, Belgium; 4 Laboratory of Tumor & Development Biology, GIGA-Cancer, University of Liège, Belgium; 5 Laboratory of Image Synthesis and Analysis (LISA), Faculty of Applied Sciences, ULB, Brussels, Belgium; 6 DIAPATH, Center for Microscopy and Molecular Imaging (CMMI), Gosselies, Belgium; 7 Department of Pathology, Erasme University Hospital, Université Libre de Bruxelles, Belgium

## Background

In the field of digital pathology and biomedical research, there is a strong need for efficient tools to build pathology atlases and to foster collaboration between researchers, pathologists (e.g. for inter-observer concordance studies) and computer scientists (e.g. for development and extensive validation of novel computer vision algorithms). Although many efforts have been made in virtual microscopy and telepathology in the recent years [1-4] , many of the resulting frameworks are not fully web-based therefore limiting collaboration, or they are vendor-dependant therefore limited in terms of supported image formats, or they use proprietary modules that prevent cross-browser compatibility and seamless execution on mobile devices, or they have restricted functionalities (e.g. images can only be annotated manually with image-level tags or fixed markers), or their design limits their application domain (e.g. education only, or disease-specific). In this paper, we present a general-purpose, rich internet application using recent web technologies and integrating various open-source tools, standards and generic algorithms for remote visualization and collaborative annotation of digital slides.

## Material and methods

### General architecture design

Our application follows a representational state transfer (REST) architecture style that structures database resources and that standardizes communication interfaces. In such a setting, each resource can be referenced by a uniform resource locator (URL) and they can be located at different physical sites and updated/deleted if necessary. By following these programming guidelines, we defined a RESTful JSON application programming interface (API) to allow communication between servers and clients.

On the server-side, our underlying data model allows to create multiple projects, where each project corresponds to a specific study or experiment. A project is described by a list of authenticated users with permission rights, a list of digital slide images, an ontology definition with domain-specific, user-defined, vocabulary terms, and annotations (regions of interest) associated to digital slides and drawn by users. All project data are stored in a spatial, relational database (PostgreSQL with PostGIS extension). The core of our application uses the Grails framework based on Spring, with Groovy dynamic programming language for Java, and Hibernate framework with its spatial extension for object/relational mapping.

On the client-side (i.e. the Web client), the source code is based on model-view-controller design patterns and it communicates directly through the API to visualize and edit resources. Data can also be retrieved or updated by third-party computer programs through the API.

### Visualization tools

In order to visualize whole-slide images at multiple resolutions in traditional web clients, we implemented the web interface with fully Javascript interfaces and libraries (including JQuery, Backbone.js and Twitter Bootstrap components). Visible parts of high-resolution images are delivered through distributed image tile servers (using IIPImage system) that supports TIFF and JPEG2000 image formats, and it was combined with the OpenSlide library to further support various digital slide image formats (Aperio SVS, Hamamatsu VMS, 3DHistech Mirax, ...). Additional caching mechanisms are implemented in-between the image servers and clients (using Varnish library) to speed up the delivery of the most frequent data.

### Annotation and collaboration tools

**Figure 1:**
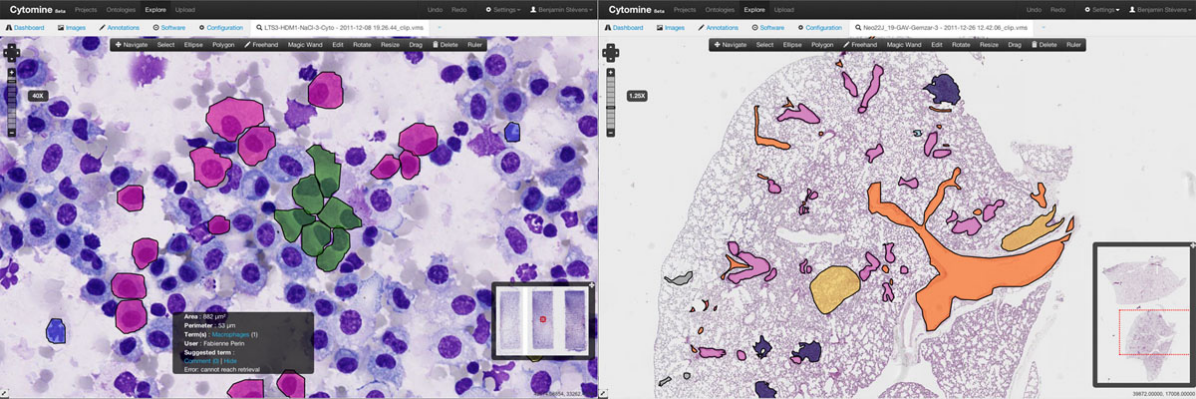
**Viewing and annotating whole-slide images** Exploration and annotation of whole-slide cytology (left) and histology (right) images from experimental mice lungs. Colors correspond to different ontology terms associated to annotations.

In addition to remote visualization capabilities, each user of a given project can create and edit his own layer of annotation geometries (e.g. polygons, ellipses, rectangles, or freehand drawings) drawn on top of digital slide images, and visualize annotations created by others associated to the current project, using the OpenLayers library, as illustrated by Figure 1. Each region of interest can be associated to one or multiple term(s) from a structured vocabulary defined on-line by the users of each project. Within a given project, relational queries are used to filter annotations based on image names, user names, and/or ontology terms, so that annotation galleries (with cropped image regions) and statistics can be easily gathered and visualized, hence facilitating the shaping of pathology atlases. Furthermore, to ease collaboration between pathologists, an e-mailing mechanism allows sharing and discussing annotations, and a communication mechanism allows one user to follow another user's observation paths in real-time through the Internet.

### Image processing and retrieval algorithms

We implemented image processing routines and a recent content-based image retrieval (CBIR) algorithm to speed up the exploration and annotation of digital slides. The image processing routines are based on ImageJ/FIJI plugins and include various image filtering operations (e.g. binarization, splitting color channels, and color deconvolution) that can be applied on-the-fly on image tiles to ease image inspection, and adaptive thresholding operations that can be used to semi-automatically draw annotation geometries around objects of interest. The CBIR algorithm uses random subwindow extraction and vectors of random tests on raw pixel values [5]. It is used to search visually similar annotations and automatically suggests ontology terms through an average voting scheme based on computed image similarities with cropped images of previously indexed annotations. We implemented the CBIR algorithm using an efficient key-value store based on hash tables (using Kyoto Cabinet or Redis NoSQL databases).

## Results and discussion

### Results

Our application runs in any popular web browsers and on mobile devices without the need for proprietary browser add-ons. It has been used for one year by our collaborators from two geographically distant locations through the Internet. About one thousand whole-slide images of lung cancer studies (corresponding to roughly 1TBytes of data) acquired by two slide scanners (Olympus VS100 with 20X magnification and Hamamatsu Nanozoomer 2.0 with 40X magnification) have been uploaded. These include Hematoxylin&Eosin (H&E) stained histology images of experimental mice, and bronchoalveolar lavage (BAL) cytology images. Three ontologies describing various tissue types (e.g. bronchus, blood vessel, cartilage, adenocarcinoma, nodular lymphoid hyperplasia,. ..) and various cell types (e.g. squamous epithelial cells, macrophages, eosinophils, neutrophils, mucosecreting cells, ciliated bronchial cells,...) were defined and used by seven users (pathologists, pneumologists, and technicians) to annotate more than five thousand regions of interest.

**Figure 2:**
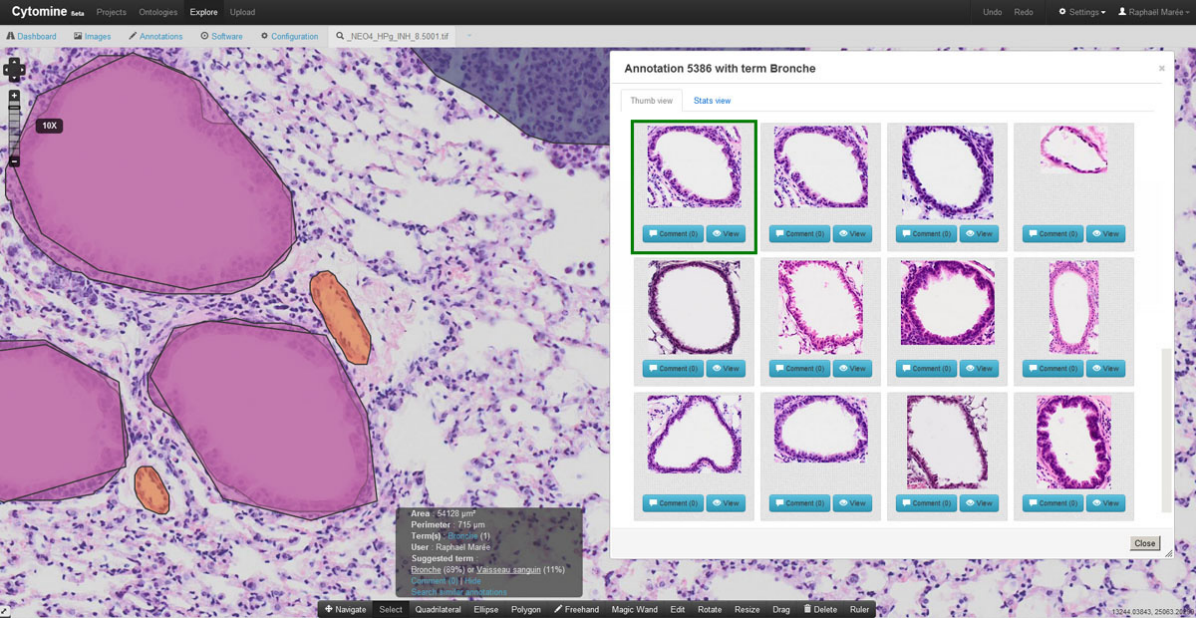
**Automatic ontology term suggestion** Once a user draw an annotation geometry, our CBIR algorithm is executed to retrieve the most similar annotations based on visual image content and suggest automatically an ontology term using average voting. The process is illustrated here with a bronchus of a lung tissue where the ten first retrieved annotations are bronchus somehow similarly oriented.

Once a user has drawn an annotation geometry on an image, our CBIR algorithm automatically suggests ontology terms in roughly 500 ms by searching visually similar regions of interest in the database of all users' annotations, as illustrated by Figure 2. Automatic term suggestions match the ground-truth (manually associated vocabulary terms) in roughly 70% for lung H&E histology images (n=1509), and 89% for lung BAL cytology images (n=675), using the same algorithm parameters for both image types as in [5] (ie. 1000 random subwindows encoded in HSV colorspace, 10 vectors of random tests, and 30 binary tests per vector). Inspection of confusion matrices reveals that per class recognition rates vary from 0% (for mucosecreting cells often misclassified as ciliated bronchial cells) to 95% (for macrophages and polynuclear neutrophils) in our cytology images, and from roughly 40% (for nodular lymphoid hyperplasia often misclassified as adenocarcinoma, and blood vessels often misclassified as bronchus) to 95 % (for bronchus and red-blood cells) in our histology images.

### Discussion

Although the amount of data our software is already dealing with is rather large, it is expected that the wider adoption of digital acquisition equipments will generate much larger datasets. The design of our software allows its scaling to larger sets of images as most of the components (e.g. image servers, and image retrieval algorithm) can be distributed on multiple machines. It is also important to note that the architecture allows local configurations, ie. images and data have not to be stored on a central, external, server but they can remain on servers at local institutions, therefore ensuring confidentiality and local administration. It is worth noting that although we do not rely on latest standard definitions in digital pathology ([1]), we plan to extend our software to support these standards once they will be implemented in the field. In the future, our architecture will also allow us to add new image formats without affecting the source code of the core application.

Regarding our preliminary evaluation with the CBIR algorithm, our results are promising for automatic term suggestion but further validation has to be conducted. Indeed, recognition rates for less frequent object types are lower, stressing the need for more manual annotations with respect to object types (e.g. mucosecreting cells were six times less frequently annotated than ciliated bronchial cells), and acquisition protocols (such as color stainings).

## Conclusions

The proposed web software is generally applicable and its methodological choices open the door for large-scale distributed and collaborative image annotation and exploitation projects. Future work includes the integration and validation of general-purpose machine learning techniques to further facilitate annotation and quantification of specific visual phenotypes and to support their meta-analysis. We also plan to extend our framework to other types of multidimensional imaging data related to other diseases or biological processes, and we also intend to adapt its use for education purposes.

## Competing interests

The author(s) declare that they have no competing interests.

## Authors' contributions

RM, BS, and LR contributed to the general design of the software. BS and LR carried out most of the software programming work. NR, DC, XML and IS have been involved in functional specifications as well as acquisition and annotation of imaging data. RM coordinates the study and drafts the manuscript. All authors read, edited, and approved the final manuscript.
